# Proportion and Reasons for Clozapine Discontinuation: A Systematic Review and Meta-Analysis

**DOI:** 10.1093/schizbullopen/sgag016

**Published:** 2026-04-30

**Authors:** Paul McLaughlin, Róisín McManus, Oisin Conaty, Faisal Al-Harbi, Fiona Gaughran, Brian O’ Donoghue, John Lally

**Affiliations:** UCD Centre for Psychosis Research, School of Medicine, University College Dublin, Dublin, Ireland; St John of God’s, Celbridge, Co Kildare, Ireland; UCD Centre for Psychosis Research, School of Medicine, University College Dublin, Dublin, Ireland; DETECT Early Intervention in Psychosis Service, Dublin, Ireland; Department of Psychiatry, St Vincent’s Hospital Fairview, Dublin, Ireland; Department of Psychosis Studies, Institute of Psychiatry, Psychology and Neuroscience (IoPPN), King’s College London, London, United Kingdom; National Psychosis Unit, South London and Maudsley NHS Foundation Trust and Institute of Psychiatry, Psychology and Neuroscience, King’s College London, 16 De Crespigny Park, London SE5 8AB, United Kingdom; UCD Centre for Psychosis Research, School of Medicine, University College Dublin, Dublin, Ireland; Department of Psychiatry, St Vincent’s University Hospital, Dublin, Ireland; UCD Centre for Psychosis Research, School of Medicine, University College Dublin, Dublin, Ireland; Department of Psychiatry, St Vincent’s Hospital Fairview, Dublin, Ireland; Department of Psychosis Studies, Institute of Psychiatry, Psychology and Neuroscience (IoPPN), King’s College London, London, United Kingdom

**Keywords:** treatment-refractory, psychosis, cessation, clozapine, adverse effects

## Abstract

*Background and Hypothesis:* Clozapine is the only recommended medication for treatment-resistant schizophrenia. Understanding reasons for clozapine discontinuation is important, as once clozapine is stopped, treatment options are limited.

*Study Design:* We conducted a systematic review and proportional meta-analysis of observational studies to identify the prevalence of clozapine discontinuation and to identify reasons for clozapine discontinuation. We systematically searched PubMed and Scopus databases from 1988 until November 2025. We included English language observational studies which reported on clozapine-treated cases and reasons for clozapine discontinuation. Study quality and risk of bias were assessed using the National Heart, Lung and Blood Institute Study Quality Assessment Tool.

*Study Results:* Forty-five studies met inclusion criteria. The pooled proportion of clozapine discontinuation was 34% (95% CI, 29%-40%, *n* = 8136, 41 studies (*n* = 22 323), *I*^2^ = 98.43%). The pooled proportion of discontinuation in the total population due to non-adherence (all reasons) was 11% (95% CI, 8%-16%, *n* = 1418, 34 studies, *I*^2^ = 97.27%); side effects 11% (95% CI, 8%-13%, *n* = 2754, 40 studies, *I*^2^ = 96.12%); and ineffectiveness, 7% (95% CI, 5%-9%, *n* = 1355, 31 studies, *I*^2^ = 96.26%).

*Conclusions:* The most common reasons for clozapine discontinuation are non-adherence and side effects. By identifying and addressing these issues, clozapine continuation and thus clinical outcomes can be optimized. Lack of consistency in reporting reasons for discontinuation may indicate incomplete representation of reasons and could affect the findings of our study.

## Introduction

Treatment-resistant schizophrenia (TRS) occurs in approximately 30% of people with schizophrenia.^1,2^ Clozapine is the only recommended medication for TRS, demonstrating improvement across multiple domains, including positive symptoms,^3,4^ hospital admissions,^5,6^ suicidality,^7,8^ aggression and substance use,^9^ as well as reductions in overall mortality.^8^

Despite clozapine’s effectiveness, it remains underutilized^10^ with a range of clinician and patient factors identified as restricting its use.^11,12^ Clinicians’ perception that patients will not tolerate side effects^13^ and their own lack of experience in managing side effects^14^ are highlighted as barriers.

Despite clozapine’s clinical benefits, people still stop taking it, albeit less so than with other antipsychotics, with a meta-analysis of non-randomized cohort studies, showing clozapine had lower all-cause discontinuation than non-clozapine antipsychotics.^6^ Nevertheless, clozapine discontinuation is a clinical reality, with approximately 40% of patients discontinuing it within 2 years of initiation.^15–17^ This in turn leads to relapse of symptoms,^18^ increased hospitalization, and poorer overall outcomes,^4,19,20^ including increased all-cause mortality, particularly within the first year after discontinuation.^21^

Clozapine discontinuation related to side effects is not only due to life threatening ones such as agranulocytosis, myocarditis, cardiomyopathy, or ileus, but often is in the context of other impairing or distressing adverse effects such as weight gain, sedation, hypersalivation, constipation, tachycardia. The prevention, recognition, and management of the more benign, yet troublesome side effects has the potential to maintain clozapine treatment for patients. However, the relative impact of specific side effects on discontinuation remains unclear.

Given the critical role of clozapine in managing TRS, understanding reasons for clozapine discontinuation is important, as once clozapine is stopped, treatment options are limited. The type and magnitude of clozapine adverse effects associated with discontinuation remain unclear. Identification of reasons for clozapine discontinuation can be helpful for clinicians and patients in shared decision making regarding treatment, while informing strategies to improve adherence and optimize patient outcomes. We conducted a systematic review of observational studies reporting reasons for clozapine discontinuation, with a specific focus on adverse effects as a cause of clozapine discontinuation. Our aim was to systematically review observational studies reporting clozapine discontinuation to estimate the pooled prevalence of clozapine discontinuation using a meta-analytical approach and to examine sources of heterogeneity. Longitudinal cohort studies, with longer follow-up, naturalistic settings, and potentially larger sample size than in clinical trials, are more suited to establish discontinuation rates and to identify reasons for discontinuation. Our goal was to inform patients and clinicians about the prevalence of different reasons for discontinuation and rates of common clozapine adverse effects associated with discontinuation.

## Methods

The protocol of this systematic review was registered in PROSPERO (CRD4-PROSPERO 2024 CRD42024584477), and its details are available at: http://www.crd.york.ac.uk/prospero/

The review was conducted in accordance with the Meta-analyses of Observational Studies in Epidemiology guidelines^22^ and reported in accordance with the Preferred Reporting Items for Systematic Reviews and Meta-Analyses (PRISMA) statement.^23^ A PRISMA checklist is provided in Table S1.

### Inclusion and Exclusion Criteria

We performed a literature search to identify all observational studies including prospective and retrospective cohort studies, case–control studies, and cross-sectional studies up until November 2025.

Inclusion criteria were: (1) Adult participants (>18 years, with no upper age limit) with a diagnosis of treatment resistant schizophrenia or schizoaffective disorder, (2) treatment with clozapine, (3) reported reasons for clozapine discontinuation, and (4) were English language studies.

The longest follow-up duration or the largest population study was included when studies reported on the same or overlapping populations.

Exclusion criteria were: (1) Non-English language studies; (2) Randomized controlled trials (RCTs) were excluded, as discontinuation in trials may be influenced by trial procedures and eligibility criteria; (3) Conference proceedings but authors were contacted to ascertain whether the data had been published in a peer-reviewed journal; (4) Review articles were excluded, but any eligible studies included in the respective review will be included in the current study; (5) Case series, case reports, editorials; (6) Studies were excluded that reported treatment discontinuation rates attributable only to specific adverse effects (eg, neutropenia/agranulocytosis, sedation, or weight gain) but did not report overall clozapine discontinuation rates or all-cause discontinuation due to adverse effects. In these studies, discontinuation data were presented only for individual side effects rather than as a comprehensive measure of clozapine cessation related to adverse events. Consequently, these studies did not allow for evaluation of overall clozapine discontinuation attributable to adverse effects and were therefore excluded from analyses examining total treatment discontinuation.

### Information Sources and Searches

Two independent reviewers (R.M., J.L.) performed an electronic search using PubMed/Medline and Scopus, with no publication date restrictions (until November 2025). The search terms are provided in Table S2.

In addition, the reference lists of the retrieved articles and relevant review articles were examined for cross-references. When necessary, corresponding authors were contacted to clarify study eligibility and/or acquire additional data.

### Study Selection

Two reviewers (R.M., P.M.) independently screened all articles against the inclusion criteria. All publications identified by the search were reviewed at the title and abstract level by 2 researchers (R.M., P.M.). Duplicate articles were removed. Articles deemed viable were cross-checked by authors to ensure that they meet the inclusion criteria. Any discordance throughout this process was resolved by arbitration with a third reviewer (J.L.).

In addition, the reference lists of the retrieved articles and relevant review articles were examined for cross-references. When necessary, corresponding authors were contacted to clarify study eligibility and/or acquire additional data.

Both reviewers then extracted information from relevant articles independently. A data extraction sheet was developed based on the pre-defined outcomes, and relevant data were extracted onto this sheet.

### Data Extraction

Data were collected in a predefined collection form incorporating study characteristics (first author name, year of publication, study design (eg, prospective, retrospective), sample size), baseline characteristics (gender, age), clozapine characteristics (mean clozapine dose before clozapine discontinuation, mean clozapine treatment duration), the number of cases undergoing clozapine discontinuation, reasons for clozapine discontinuation (eg, side effects, non-adherence, ineffectiveness, unknown, death, concomitant medical issues), and specific side effects associated with discontinuation (hematological, cardiovascular, gastrointestinal, glucose intolerance, weight gain, sedation, hypersalivation, hypotension). Due to variability in reporting across studies, discontinuation categories were harmonized into broader groups (eg, non-adherence, side effects, ineffectiveness). Where overlapping terminology was used (eg, patient decision, refusal of blood monitoring), these were grouped under non-adherence unless explicitly defined otherwise. We acknowledge that some overlap between categories is likely. Two of the authors (R.M. and P.M.) independently extracted the data. Any disagreements regarding the data extraction were resolved by consensus with the senior author (J.L.).

### Primary/Secondary Outcome

The primary outcome was the prevalence of clozapine discontinuation. Secondary outcomes were: (1) Prevalence of clozapine discontinuation due to: (a) Side effects (b) non-adherence, (c) ineffectiveness and (2) in those who discontinued clozapine, the prevalence of discontinuation due to: (a) Side effects (b) non-adherence, (c) ineffectiveness.

Discontinuation rates due to mortality, individual side effects, and categories of side effects are reported. Deaths were included as discontinuation events only where studies explicitly reported death as the reason for treatment cessation.

### Data Synthesis

A meta-analysis of proportions was conducted using STATA 18 using the meta proportions command. The logit-transformed proportion was used in the meta-analysis as there was a variance in the proportion of discontinuation across the different studies, and this method is recommended if the proportions are not close to 0.5.

We performed a random effects proportional meta-analysis to identify the pooled prevalence of clozapine discontinuation and specific reasons for discontinuation and the corresponding 95% CIs. Heterogeneity was assessed using both the *I*^2^ statistic and the *Cochran’s Q* test, with *P* < .05 indicating significant heterogeneity of the pooled results.

Sources of heterogeneity were explored with meta-regression and subgroup analyses. Random-effects meta-regression analyses were conducted with mean age, gender, sample size, mean clozapine dose, and time to discontinuation (equivalent to duration of clozapine use).

Subgroup analyses were also conducted based on region of study (Europe vs North America vs Rest of World), year of study (median) (<2011; ≥2011), sample size (median) (<130.5, ≥130.5), study design (prospective vs retrospective vs case–control), mean age (<38.4 vs ≥38.4 years), clozapine dose (<350 vs ≥350 mg/day), duration of clozapine treatment (≤6 vs >6 months), and study quality assessment score (good vs fair vs poor) to explore possible sources of heterogeneity between studies.

We assessed publication bias with a visual inspection of funnel plots and quantitative testing through the use of Begg–Mazumdar^24^ and Egger bias tests,^25^ with a *P* value <.05 suggesting the presence of bias.

### Data Analysis

Data were analyzed as frequency and percentages for categorical variables and mean and SD for the continuous variables.

Descriptive analyses (frequencies, percentages, means, and SD) on demographic data and the individual reasons for discontinuation were performed as appropriate. Results from included studies were narratively synthesized and summarized.

### Risk of Bias and Quality Appraisal in Individual Studies

The Study Quality Assessment Tool developed by the National Heart, Lung and Blood Institute (NHLBI) was used to assess the risk of bias and quality of the studies. The tool includes items for evaluating potential flaws in study methods or implementation, including sources of bias, confounding, study power, the strength of causality in the association between interventions and outcomes, and other factors. Quality reviewers could select “yes,” “no,” or “cannot determine/not reported/not applicable” in response to each item on the tool. Reviewers then judged each study to be of “good,” “fair,” or “poor” quality. In general terms, a “good” study has the least risk of bias, and results are considered to be valid. A “fair” study is susceptible to some bias deemed not sufficient to invalidate its results. A “poor” rating indicates significant risk of bias. The NHLBI tool was used independently by 3 reviewers (P.M., O.C., F.E.H.). Disagreements were solved by consensus with a fourth reviewer (J.L.).

## Results

**Figure 1:**
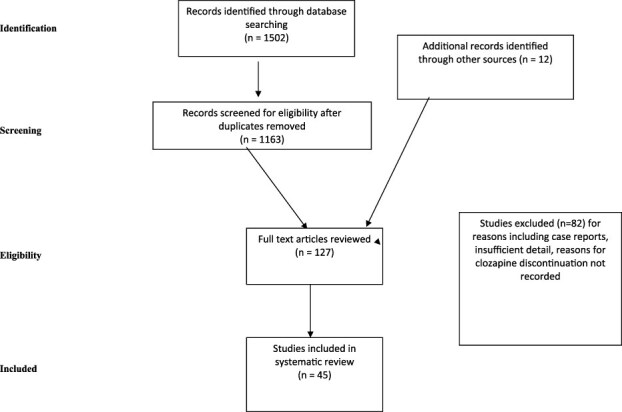
Flowchart of Studies Selection.

The study selection process, search results, and reasons for exclusion are given in Figure 1.

The initial search yielded 1502 articles. After removal of duplicates, the titles and abstracts of 1163 articles were screened, with 1046 articles excluded, and an additional 10 articles identified through other sources. A total of 127 full text articles were screened for eligibility with 82 articles excluded with reasons (Figure 1), leaving 45 studies included in the systematic review. Citations within each article were included as an additional source of references.

### Study and Participant Characteristics

A total of 45 studies were included in this systematic review, including 37 retrospective studies,^26–29^ 8 prospective studies,^15,30–36^ and 1 case–control study.^37^ The included studies were all in English and were published between 1988 and 2025.

Forty percent (*n* = 18) of studies were from Europe, 27% (*n* = 12) from North America, and 33% (*n* = 15) from the rest of the world. The sample size ranged from 10^38^ to 8263,^39^ and 25 studies had sample sizes of over 100.

Sixty-eight percent of patients were male, and 94% (*n* = 30) of studies (*n* = 32 studies reporting sex) had a majority of male participants. The mean age of the total population was 38.4 (SD = 11.7) years (range 19.5-74.2 years).

The mean duration of clozapine use was 2.1 (SD = 1.6) years (range 0.22-5.9 years). The mean clozapine dose at time of discontinuation was 302.9 mg (SD = 109.9) (range 83-503 mg).

Full details of the included studies are summarized in Table S3.

### Quality of Included Studies

Using the NHLBI study quality assessment tool, 42% (*n* = 19) of studies were assessed as good (*n* = 19 [*n* = 5 prospective studies,^15,30,32–34^  *n* = 13 retrospective studies,^16,26,39–49^ and *n* = 1 case–control^37^]), 33% (*n* = 15) as fair (*n* = 15 studies [*n* = 1 prospective studies^35^ and *n* = 14 retrospective studies^27–29,36,50–59^]), and 24% (*n* = 11) as poor (*n* = 11 studies [*n* = 1 prospective studies^31^ and *n* = 10 retrospective studies^38,60–68^]). Full details of the assessment of the quality of the included studies are summarized in Table S4.

### Meta-Analysis

**Figure 2:**
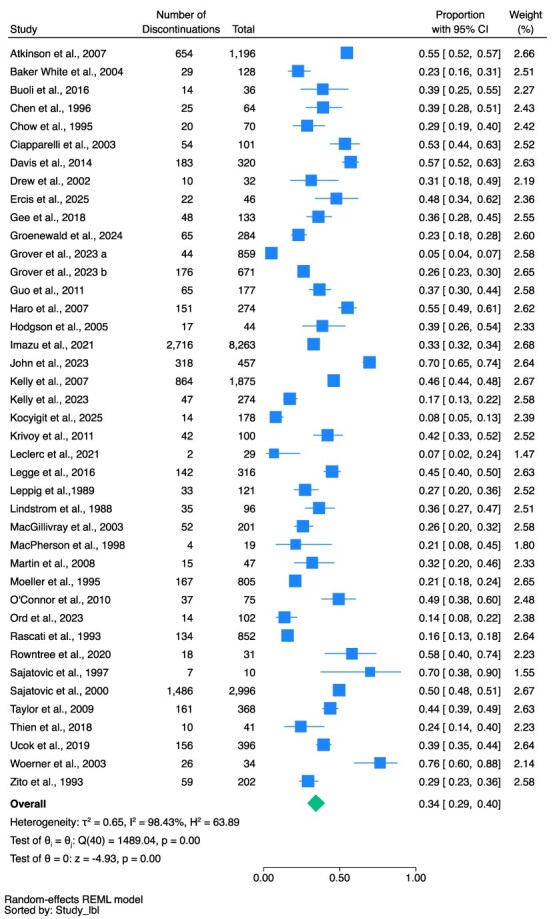
Total Proportion Who Discontinued Clozapine (All Cause).

The pooled proportion of clozapine discontinuation was 34% (95% CI, 29%-40%, *n* = 8136, 41 studies [*n* = 22 323], *I*^2^ = 98.43%) (Figure 2). The range of clozapine discontinuation rates was from 5%^61^ to 76%.^35^

**Figure 3:**
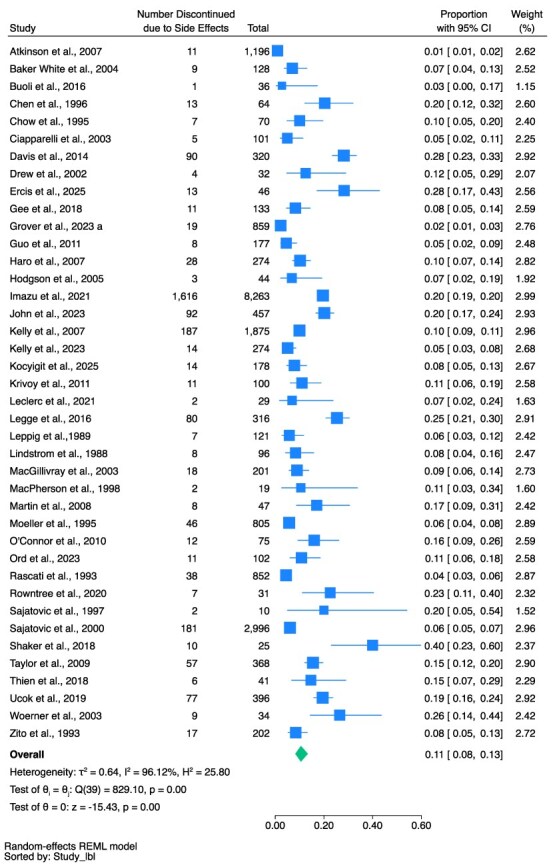
Discontinued Due to Side Effects as a Proportion of Total People Prescribed Clozapine.

**Figure 4:**
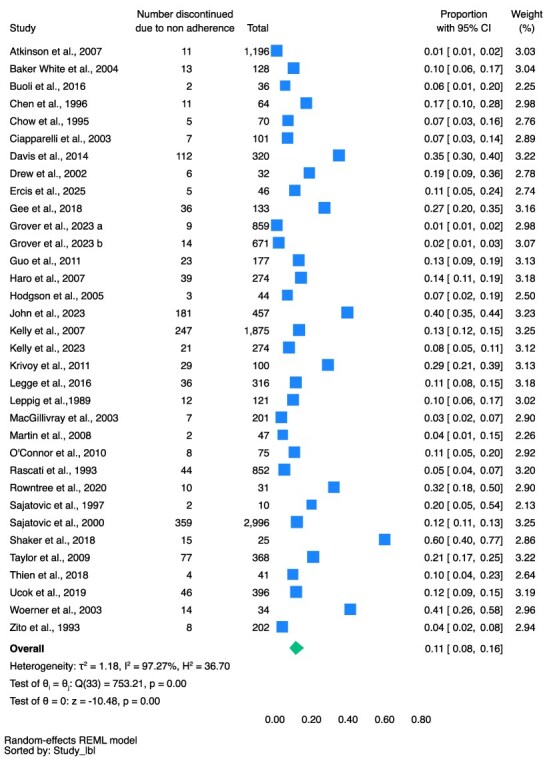
Discontinued Due to Non-Adherence (All Reasons) as a Proportion of Total People Prescribed Clozapine.

**Figure 5:**
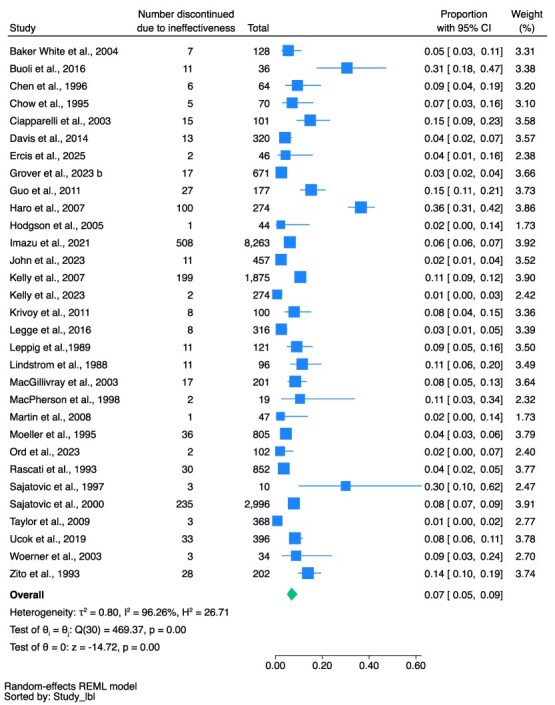
Discontinued Due to Ineffectiveness as a Proportion of Total People Prescribed Clozapine.

The pooled proportion of discontinuation in the total population due to side effects was 11% (95% CI, 8%-13%, *n* = 2754, 40 studies, *I*^2^ = 96.12%) (see Figure 3); due to non-adherence (all reasons) was 11% (95% CI, 8%-16%, *n* = 1418, 34 studies, *I*^2^ = 97.27%) (see Figure 4); due to ineffectiveness was 7% (95% CI, 5%-9%, *n* = 1355, 31 studies, *I*^2^ = 96.26%) (see Figure 5).

In those who discontinued clozapine, the pooled prevalence of discontinuation due to: Side effects was 34% (95% CI, 29%-39%, *n* = 2845, 43 studies, *I*^2^ = 92.23%) (Figure S1); due to non-adherence (all reasons) was 35% (95% CI, 2%9-42%, *n* = 1642, 37 studies, *I*^2^ = 94.24%) (Figure S2); due to ineffectiveness was 18% (95% CI, 13%-25%, *n* = 1372, 34 studies, *I*^2^ = 96.72%) (Figure S3).

### Subgroup Analyses and Predictors of Clozapine Discontinuation

The subgroup analyses are summarized in Table S5.

Studies of good quality (*n* = 18) did not have a significantly higher pooled prevalence of clozapine discontinuation (39.9% [95% CI, 31.7%-48.7%]) compared to studies of fair (*n* = 13; 34.5% [95% CI, 26.7%-43.3%]) and poor quality (*n* = 10; 24% [95% CI, 14.3%-37.4%]) (*Q* = 1489.04, *P* = .136) (*I*^2^ = 98.43%).

There was no significant difference in pooled prevalences of clozapine discontinuation across different study designs (retrospective, prospective, case–control), region of study (North America, Europe, Rest Of World), median sample size (<130.5 or ≥130.5), median year of study (<2011 or ≥2011), mean age (<38.4 vs ≥38.4 years), clozapine duration pre discontinuation (<6 or ≥6 months), and clozapine dose (<350 vs ≥350 mg/day).

The separate meta-regressions are presented in Table S6. Briefly, no significant moderators of clozapine discontinuation were identified. A longer mean duration of clozapine use was not significantly associated with a higher pooled prevalence of clozapine discontinuation (*n* = 17 comparisons) (β = 0.0792, 95% CI, −0.0243 to 0.1826, *P* = .134).

Neither the Begg Mazumdar (Kendall’s Tau =13.0, *z* = 0.14, *P* = .889) nor the Egger test (bias 0.38, *P* = .698) indicated the presence of publication bias. A visual inspection of the funnel plot revealed some asymmetry (Figure S4).

### Descriptive/Narrative Synthesis

Rates of clozapine discontinuation in the total population due to adverse effects ranged from 0.9%^26^ to 40%,^27^ due to non-adherence from 0.9%^26^ to 60%,^27^ and due to ineffectiveness from 0.7%^34^ to 36.5%.^33^ In clozapine, discontinuers reported rates of discontinuation due to adverse effects were 7%^30^ to 100%,^44,63^ due to non-adherence was from 8.0%^62^ to 75.5%,^66^ and due to ineffectiveness was from 1.9%^37^ to 79%.^30^

Blood refusal accounted for 3.5%^46^ to 20%^38^ of discontinuations in the total population (*n* = 5 studies), with average discontinuation rates of 10.7% due to blood refusal in all cases; and 18.6% (*n* = 5 studies) (range 7.8%^46^ to 28.6%^38^) of discontinuation in clozapine discontinuers.

Patient decision to stop clozapine accounted for 2.1%^62^ to 13.0%^32^ of all discontinuations with a mean rate of 6.9% (*n* = 10 studies). In clozapine discontinuers, a patient decision to discontinue clozapine accounted for 7.9%^43^ to 40%^66^ of discontinuations (*n* = 10 studies), with a mean rate of 22.1% (*n* = 10 studies) in those who discontinued clozapine.

### Side Effects

In those who discontinued clozapine for any reason, discontinuation due to hematological side effects (*n* = 22 studies) ranged from 4.0%^27^ to 25%,^65^ for sedation (*n* = 13 studies) ranged from 1%^39^ to 48%,^42^ for cardiac side effects (*n* = 10 studies) ranged from 1.5%^39^ to 60%,^28^ for hypotension (*n* = 10 studies) ranged from 0.9%^43^ to 25%,^65^ for gastrointestinal (*n* = 4 studies) ranged from 2.7%^39^ to 14.2%,^59^ hypersalivation (*n* = 6 studies) ranged from 0.3%^43^ to 28%,^42^ glucose intolerance (*n* = 4 studies) ranged from 1.3%^29^ to 10%,^52^ weight gain (*n* = 4 studies) ranged from 0.9%^43^ to 6.7%.^56^

Death (regardless of whether the cause was attributed to clozapine) was recorded as a reason for discontinuation in 11 studies (*n* = 181 deaths).^16,26,37,43,46–48,54,57,58,68^ In the 10 studies that reported discontinuation due to death, from the total population of clozapine users, mortality rates ranged from 1% (*n* = 12 cases)^26^ to 18.7% (*n* = 14 cases)^58^ with an average rate of 1.9% (*n* = 162) (*n* = 10 studies, total *n* = 8609). A retrospective study identified a rate of 10% (*n* = 19) discontinuation due to death in those who discontinued clozapine.^48^ From all deaths reported (*n* = 181), the cause of death was attributable to clozapine in 4 cases^16,68^ and possibly attributable to clozapine in a further 7 cases.^26^ See Table S7 for causes of death as documented in respective studies. In those who discontinued clozapine in those 11 studies in which deaths were reported, an average of 4.3% (*n* = 181) of discontinuations were due to death (*n* = 11 studies, total *n* = 4254) ranging from 2%^46^ to 38%^58^ of those who discontinued clozapine.

## Discussion

To the best of our knowledge this is the first systematic review and meta analyses to assess reasons for clozapine discontinuation. We identified that 34% of people prescribed clozapine discontinue it over a follow-up period averaging 2.1 years. Clozapine is the only approved medication in TRS, so this reduces treatment options. Further, clozapine discontinuation can cause a rapid deterioration in symptoms,^18,69^ longer admission episodes,^70^ and is associated with poorer functioning.^26,71^

The most common reasons for clozapine discontinuation are non-adherence and side effects. By identifying and addressing these issues, clozapine continuation can be optimized, and the poorer outcomes associated with clozapine discontinuation averted.^20^

Our finding that non-adherence (11%) was a common reason for clozapine discontinuation is consistent with several well-designed cohort studies.^16,66^ However, there is a paucity of reporting of reasons for non-adherence, which could relate to patient, clinical, or service level factors. We were able to identify specific reasons for non-adherence in a limited number of studies, from 7.9%^43^ to 40%^66^ of all discontinuations due to patient decision, while non-compliance with mandatory blood monitoring accounted for between 3.5%^46^ to 20%^38^ of the total study populations, offering some granularity to the finding of non-adherence. The finding that approximately 10.7% of discontinuations were attributable to blood monitoring refusal (*n* = 5 studies which recorded this) highlights this as a key modifiable barrier and supports recent international recommendations advocating for simplified monitoring protocols.^72–74^ Non-adherence to antipsychotics is common in schizophrenia with rates of 40%-50%,^75^ higher than that observed in our study. Non-adherence due to unspecified reasons and adverse effects are the most commonly reported reasons for interruption of clozapine treatment. Preventing and managing side effects when they occur may lead to reductions in non-adherence.

### Adverse Effects and Clozapine Discontinuation

We found that adverse effects were another common reason for discontinuation at 11%, similar to those rates reported in a number of cohort studies.^26,34,38,43,57^ Despite side effect burden, clozapine is associated with reduced all-cause mortality compared to schizophrenia cases treated with non-clozapine antipsychotics or no antipsychotics.^8,76–79^ From the early stages of clozapine treatment, it is possible to assess for reversible and treatable adverse effects. Engagement with patients, asking about and responding to reports of side effects, is important to reduce the likelihood of clozapine discontinuation.

Slower clozapine titration is proposed to reduce the risk of early onset adverse events and to enhance clozapine safety,^80^ although we could not assess that in this study. Patient education about potential side effects should be offered pre-clozapine and during the early stages of clozapine use, allowing people to make informed decisions and better cope with adverse effects should they arise. This can lead to greater understanding and awareness of how to manage certain side effects and that adverse effects can be treated, for example, prescribing stimulant laxatives to prevent or treat constipation,^81^ using hyoscine hydrobromide to manage sialorrhoea,^82^ or co-commencing metformin, in conjunction with promoting a healthy lifestyle around diet and exercise, to prevent weight gain.^83,84^

There was wide variability between studies in the proportion of reported discontinuation rates that were due to adverse effects, ranging from 0.9%^26^ to 40%.^27^ In clozapine discontinuers, adverse effects such as neutropenia or agranulocytosis (range 4%^27^ to 28%,^66^  *n* = 22 studies), and sedation (range 1%^39^ to 48%,^42^  *n* = 13 studies) were the most frequently reported reasons for discontinuation due to adverse effects. The estimated rate of clozapine discontinuation due to hematological adverse effects should be interpreted with caution, as it was derived from a limited number of studies. Several large cohort studies reporting on the incidence of clozapine-associated hematological adverse effects did not meet the predefined inclusion criteria for this review^85–88^ and were therefore excluded from the analysis. Consequently, the available evidence base informing discontinuation due to hematological complications was relatively restricted. Furthermore, we were unable to clarify if discontinuation due to neutropenia was attributable to clozapine-induced agranulocytosis or clozapine-associated agranulocytosis or neutropenia (0.5-1.5 × 10^9^/L), or benign ethnic neutropenia (BEN). This is relevant in terms of future treatment options. BEN is a non-pathological condition observed in certain ethnic groups and does not confer the same infection risk as agranulocytosis. Failure to distinguish between these entities may lead to unnecessary discontinuation and reduced access to clozapine. Greater awareness of clozapine-associated neutropenia and BEN and the use of adjusted monitoring thresholds may support safe continuation or rechallenge in appropriate patients.

Variation in the pooled prevalence of side effect rates as a reason for clozapine discontinuation may relate to variations in monitoring and in implementation of mitigation strategies such as earlier recognition and dose adjustments across settings, along with different thresholds for tolerability among patients and clinicians. It may also be the case that a patient (and clinician) is more likely to continue with clozapine and tolerate or manage side effects in context of a clinical response.

A survey of clozapine prescribers exploring prescribers attitudes toward clozapine use identified that side effects and their management are a barrier to clozapine use.^14^ This study, and others, suggests professional development programs focused on managing side effects and rechallenge may promote clozapine use.^89^ Providing clinicians with education in clozapine use and the management of common adverse effects can increase clozapine prescribing rates^90^ and reduce clozapine discontinuation.^91^ Clinicians should be vigilant in screening for and managing common adverse effects which may be alleviated through dose adjustment, augmentation strategies, or supportive interventions.^92^ Such recognition and management would likely help to optimize clozapine use.

### Discontinuation Due to Ineffectiveness

A small discontinuation rate due to ineffectiveness was identified in our study, occurring in 7% of all clozapine treated cases, accounting for 18% of those who discontinued clozapine. This is a much lower rate of discontinuation than seen in non-clozapine antipsychotic RCTs, where 40% of discontinuations are due to inefficacy and 20% due to side effects.^93^ A discontinuation rate of 7% due to ineffectiveness may be an overestimate of clinical effectiveness, given that clozapine non-response in RCTs is estimated to be 40%-60%,^2,94^ although naturalistic studies identify clozapine benefits in relation to adherence and reduced discontinuation and hospitalization rates compared to non-clozapine antipsychotics.^6^ It may be that non-response is not recorded as a reason for discontinuation, for example if a patient is experiencing intolerable side effects, perhaps limiting dosing to a less effective level. However, it is also possible that this low rate reflects the lack of alternatives to clozapine, and the resultant tendency to augment clozapine rather than substitute medications should response be sub-optimal. Additionally, suboptimal response may be attributed to factors such as inadequate dosing, non-adherence, or comorbidities rather than true ineffectiveness. As such, ineffectiveness may be underreported as a primary reason for discontinuation.

Suboptimal effectiveness on clozapine is nevertheless an important potentially modifiable factor. Earlier clozapine use is associated with improved effectiveness,^95,96^ while measuring plasma clozapine concentrations to optimize clozapine concentrations and avoid suboptimal dosing, evaluation for non-adherence, and active management of mental and physical co-morbidities offer pathways to optimize the effectiveness of this potentially life changing intervention.

## Strengths and Limitations

The findings of this study should be interpreted in the context of its strengths and limitations. It was conducted by rigorously following methodological guidelines for systematic review and meta-analyses, and to our knowledge, this is the first systematic review to examine the reasons for clozapine discontinuation. By pooling discontinuation rates across observational studies, we provide the pooled prevalence rates for discontinuation which mirror real world clinical practice, with inherent diversity in patient groups, treatment settings, and management strategies. By providing specific reasons for discontinuation alongside a pooled all-cause discontinuation rate, our findings can provide guidance in relation to more specific interventions to mitigate clozapine discontinuation. This review includes both patients recently initiated on clozapine and those established on treatment, capturing discontinuation across the full treatment trajectory. While these represent distinct clinical phases (early vs longer-term discontinuation), inclusion of both enhances generalizability and reflects real-world clinical practice. However, this may contribute to heterogeneity in discontinuation rates.

Among the strengths of this meta-analysis are the large number of studies (*n* = 45) that met the inclusion criteria, and the substantial number of clozapine-treated cases (*n* = 22 348), with the majority of studies included of good to fair quality. However, most included studies were single-center, retrospective cohort studies, inherently at risk of reporting bias, and were generally at some risk of bias. The use of subgroup analyses strengthened our ability to explore sources of heterogeneity. We observed a non-statistically significant higher pooled prevalence of discontinuation in studies rated as good or fair quality (76% of all studies) compared with those of poor quality. This pattern may reflect better defined outcome assessment criteria applied in higher-quality studies, which could reduce the risk of underreporting discontinuation. Conversely, studies of lower quality may be more prone to reporting bias, incomplete follow-up, or less stringent definitions of discontinuation, leading to lower prevalence estimates.

Our study also has other limitations. First, significant heterogeneity across studies was evident; studies with different sample sizes, duration of follow-up, and reporting of reasons for discontinuation were combined. The high heterogeneity observed (*I*^2^ = 98%) likely reflects differences in healthcare systems, regulatory frameworks for clozapine monitoring, thresholds for discontinuation, and variation in clinical settings (eg, inpatient vs community care). Differences in monitoring requirements, particularly for hematological parameters, may directly influence discontinuation decisions across regions. However, in proportional meta-analysis, true heterogeneity is expected in prevalence estimates due to differences in study settings and time of study, along with the nature of proportional data which may show little variance even in small sample-sized studies^97^; in this respect, a high *I*^2^ in proportional meta-analysis may not be indicative of inconsistent data. A further contributor to heterogeneity is likely the wide variation in follow-up duration across studies, which was not systematically captured. Studies with shorter follow-up periods are less likely to capture later discontinuation events, while longer studies may reflect cumulative discontinuation over time. Pooled estimates should be interpreted as reflecting an average across differing observation periods rather than a fixed time-point estimate. Although meta-regression did not identify time to clozapine discontinuation as a significant moderator, this may reflect limited statistical power and variability in reporting. Despite heterogeneity, consistent patterns emerged, particularly the prominence of non-adherence and adverse effects as drivers of discontinuation. These findings provide clinically actionable targets for intervention and reflect real-world practice across diverse settings.

Second, the meta-analyzed studies were observational studies, the non-randomized nature of which limits the interpretation of causality. However, longitudinal cohort studies, with longer follow-up, naturalistic settings, and potentially larger sample size than in RCTs, are more suited to establish discontinuation rates and to identify precipitating or mitigating factors associated with discontinuation. However, there are limitations of naturalistic, observational data such as these, including that clinicians may be unlikely to stop clozapine treatment once started, given a perception that it is a treatment of last resort.^13^

Thirdly, the reasons for discontinuation were pooled from studies spanning nearly 30 years, and it is possible that management of clozapine use and reasons for discontinuation may have changed in that time, though we did not identify study year as a moderator of clozapine discontinuation.

Fourth, there was substantial inconsistency in reporting reasons for discontinuation, patient decision vs non-adherence vs side effects, as specific causes for discontinuation. Included studies did not typically consider the same reasons for discontinuation and did not report adverse effects in a consistent standardized manner. As studies may have used different measures to report reasons for discontinuation, in order to pool results, we combined risk estimates that have somewhat different characteristics, which could have led to some imprecision. However, since discontinuation is a clearly identifiable event, and given that a majority of included studies used a cohort design and evaluated the same population of interest, the degree of imprecision is likely low. It remains useful for future studies to standardize reporting of reasons for discontinuation. The wide range in discontinuation rates due to specific adverse effects likely reflects differences in study design (eg, incident cohorts vs prevalent users), variability in reporting practices, and whether studies focused on specific adverse events. Differences in monitoring intensity and diagnostic thresholds may also contribute. There is a need for side effects to be measured consistently across studies, perhaps with the use of Glasgow antipsychotic side effect scale for GASS-C.^98^

Fifth, few studies in this review looked at patient-reported reasons for non-adherence or discontinuation which may be additionally important to inform the design of interventions.

Further, our protocol specified adherence, side effects, and ineffectiveness as discontinuation reasons of interest. However, other causes of discontinuation were reported including, doctor decision (without it being specified if for reasons of effectiveness or intolerability, safety, or adherence concerns), administrative reasons, and death. Eleven of the studies recorded discontinuation due to death, with rates ranging from 1%^26^ to 18.7%^58^ of the total population on clozapine, with other included studies not reporting death as reason for clozapine discontinuation. Across 10 studies with a total population prescribed clozapine of 8609, 1.9% (*n* = 162) died, with only 4 cases specifically reported as having died from causes attributable to clozapine^16,68^ and a further 7 deaths possibly attributable to clozapine.^26^ The mean length of follow-up in these 10 studies was 3.1 (SD = 2.3) years, while overall it was 2.1 (SD = 1.6) years, which may be relevant to the interpretation of these findings. Overall, rates of premature mortality are lower on clozapine.^8,76^ Variation in mortality-related discontinuation may reflect differences in cohort age, physical health burden, and duration of follow-up across studies. Furthermore, our search strategy focused on terms related to discontinuation (eg, cessation, discontinuation) and adverse effects, and did not explicitly include broader terms such as non-adherence or treatment interruption. While this approach increased specificity for identifying studies reporting discontinuation outcomes, it is possible that studies describing discontinuation primarily in terms of adherence were not captured.

Sixth, few studies evaluated clozapine discontinuation in early episode schizophrenia, subgroups of considerable interest, particularly as TRS is present in up to 23% of first episode psychosis cases.^95^

Seventh, the search strategy was limited to PubMed and Scopus, and although supplemented by manual reference screening, it is possible that relevant studies indexed exclusively in EMBASE or PsycINFO were not captured; and restricting our study to the English language may limit the generalizability of our findings.

## Recommendations

Improved support for adherence including psychoeducation, proactively addressing side effects, simplifying hematological monitoring requirements^72^ and improved continuity of care are recommended. We can more robustly address and mitigate the reasons why patients are stopping clozapine due to non-adherence. Engaging in shared decision making by involving patients and their families in discussions about the benefits and risks of clozapine treatment helps build trust and empowers patients in their treatment course. We need to ensure that patients concerns are taken into account by clinicians. Our study showed that 7% discontinued due to patient decision (in studies where this was recorded). A retrospective cohort study identified that over 50% of discontinuations due to patient decisions are due to sedation.^46^ This highlights the importance of providing timely and optimal interventions for adverse effects and for studies to provide more detailed descriptions of the reasons for discontinuation.

We can look at ways to increase adherence to the medication itself. Prior to commencement of clozapine, it is important that the risks associated with clozapine are communicated in a balanced and strategic manner to patients and their families to ensure they are well-informed without causing unnecessary anxiety. It is also necessary to emphasize the benefits and inform people that time is required to assess the full clinical benefit so a longer duration of use is needed for an adequate trial. Allowing for this time would hopefully lead to a better clinical response to clozapine which in itself improves adherence.^99^

Improving patients’ knowledge, preparing them for their treatment, and highlighting the benefits of the treatment can aid adherence. We should address and solve the reasons why patients refuse to comply with the necessary blood work. The use of point-of-care-testing using capillary samples for FBCs may improve this. Regulatory revisions to clozapine hematological monitoring recommendations may aid adherence due to less intensive monitoring requirements,^72,100,101^ although this change has not yet taken place in some jurisdictions, such as the United Kingdom.

Early identification and proactive management of clozapine side effects are important interventions to mitigate discontinuation rates.^92^ The most important action is to ask. Standardized rating scales such as the GASS-C or the GASS plus the Bristol Stool Chart can open discussions and strengthen the therapeutic relationship. Some challenges for continuation are dose or concentration dependent, such as seizures and sedation. By altering timings, reducing the dose, or implementing both pharmacological or non-pharmacological mitigations, these adverse outcomes can be managed more successfully for patients.

Optimizing clozapine use, ensuring adequate dose and plasma concentrations can improve outcomes and effectiveness.^102^

Clozapine discontinuation is variably reported, with studies reporting non-adherence without specifying reason for same and others reporting discontinuation due to adverse effects, but not specifying which adverse effects. Lack of consistency in reporting may indicate incomplete representation of reasons for discontinuation and impact on interpretation of data. Hence, it would be useful for future studies to standardize reporting of reasons for discontinuation. The different study designs are prone to specific biases, and a more complete evidence base requires information from both randomized controlled trials with longer term follow-up (eg, >2 years) and observational data.

## Conclusion

This systematic review and meta-analysis provides a robust real-world report of why clozapine is discontinued, informing pathways to optimize clozapine use and mitigate discontinuation. Moreover, directions for future research and clinical interventions are provided to reduce burden of side effects and address nonadherence. Future research should seek to more consistently delineate the reasons for clozapine cessation to inform interventions to promote adherence.

## Supplementary Material

sgag016_Supplementary_Tables_and_Figures_Final_Version
