# Supplementary material for: Proportion and Reasons for Clozapine Discontinuation: A Systematic Review and Meta-Analysis
Source: Schizophr Bull Open. 2026 Apr 30;7(1):sgag016. doi: 10.1093/schizbullopen/sgag016 (PMC13213822; doi:10.1093/schizbullopen/sgag016)
Supplement: sgag016_Supplementary_Tables_and_Figures_Final_Version [file sgag016_supplementary_tables_and_figures_final_version.docx]

**Supplementary Tables & Figures**

Supplementary Table 1 - PRISMA Checklist
 **Preferred Reporting Items for Systematic reviews and Meta-Analyses (PRISMA) Checklist**

**
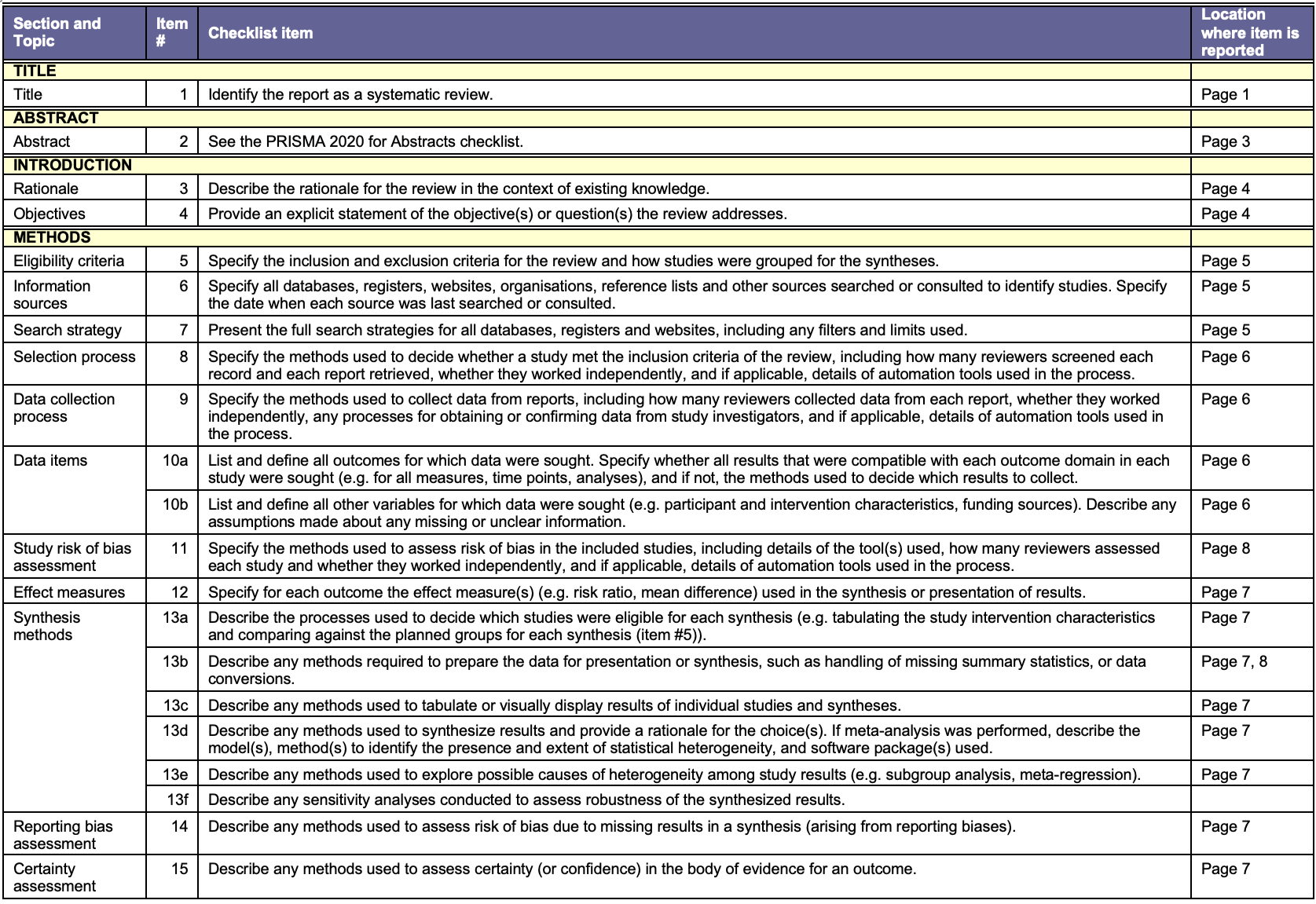
**


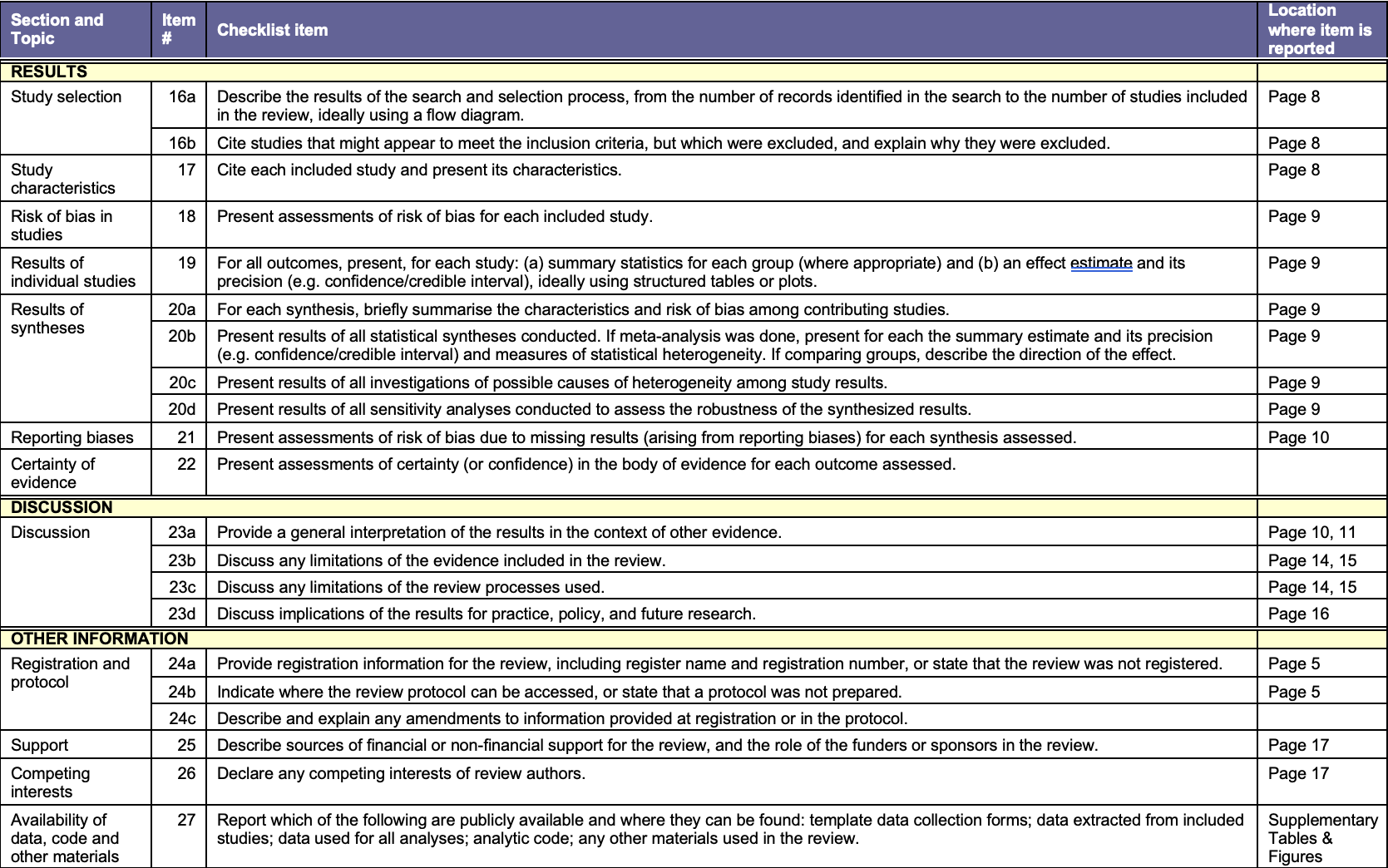


**Preferred Reporting Items for Systematic reviews and Meta-Analyses (PRISMA) for Abstract Checklist**

**
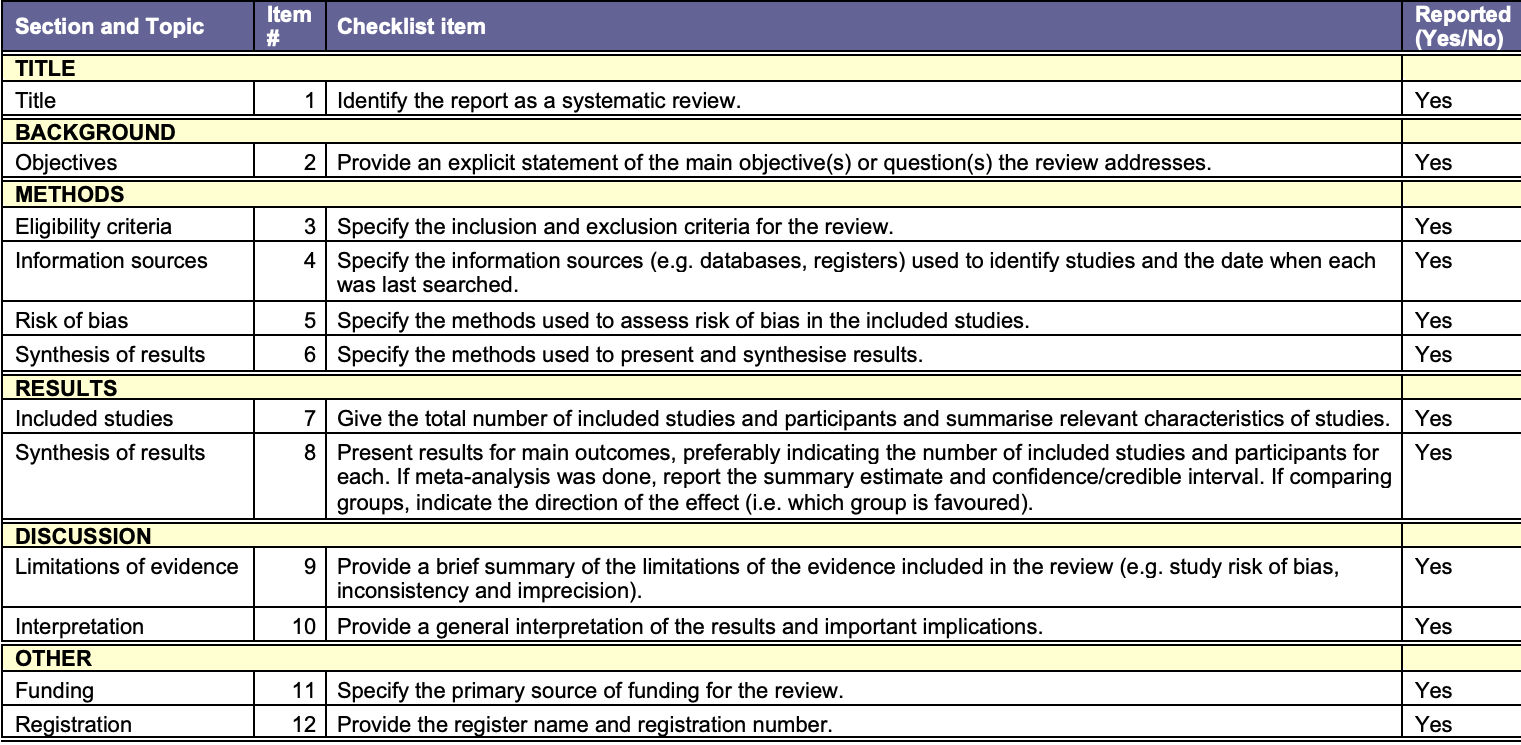
**

Supplementary Table 2 - Search Terms

| Database | Search Terms |
| --- | --- |
| PubMed | ("clozapine"[MeSH Terms] OR "clozapine"[All Fields] OR "clozapin"[All Fields] OR "clozapine s"[All Fields]) AND ("adverse effects"[All Fields] OR "side effects"[All Fields]) OR ("discontinuation"[All Fields] OR "cessation"[All Fields]) |
|  |  |
| Scopus | ( TITLE-ABS-KEY ( clozapine ) AND TITLE-ABS-KEY ( discontinuation ) OR TITLE-ABS-KEY ( cessation ) AND TITLE-ABS-KEY ( side effects ) OR TITLE-ABS-KEY ( adverse effects ) ) |

| **Supplementary Table 3 – Details of Included Studies** | | | | | | | | | | |
| --- | --- | --- | --- | --- | --- | --- | --- | --- | --- | --- |
| **Author, Year** | **Study Design** | **No. of Clozapine treated patients** | **Gender (%)** | **Age (mean (SD))** | **Mean dose of Clozapine (mg/day)** | **Duration of clozapine treatment in years (mean (SD) or median (range))** | **Clozapine discontinuation % / n** | **Reasons for discontinuation**  **% of discontinuations (n=)** | **Quality Assessment** | **Quality Assessment Score** |
| Atkinson JM, 2007 | Retrospective cohort | 1196 | Male 74.3%  Female 25.7% | 46.7 (14.9) Range 26–83 | NR | Mean 3.4 (1.8) years | 54.7% / 654 | Non compliance 31.4% (11)  Neutropenia/agranulocytosis 17.1% (6)  Intolerable adverse effects 14.3%(5)  Prescriber decision 2.9% (1)  Patient deceased 34.3% (12) | Good | 3 |
| Baker White, 2004 | Retrospective | 128 | NR | NR | 83mg | Mean 1.08 years Median 0.25 years | 23% / 29 | Failure to comply 45% (13) Unacceptable side effects 31% (9) Inadequate response 24% (7) | Fair | 2 |
| Buoli M, 2016 | Prospective  comparative cohort study | 36 | Male 75%  Female 25% | 26.72 (5.3) | 325mg | NR | 38.8% / 14 | Inefficacy 78.6% (11)  Side effects 7% (1)  Non-compliance 14% (2) | Good | 3 |
| Chen E, 1996 | Retrospective cohort | 64 | Male 71.9%  Female 28.1% | 34.5 (10.7)  Range 17-78 | 432.4mg | NR | 39% / 25 | Refused bloods 16% (4)  Side effects 52% (13)  Treatment aborted 20% (5)  Patient refusal 28% (7)  No efficacy 24% (6) | Fair | 2 |
| Chow EW, 1995 | Prospective cohort | 70 | Male 62%  Female 38% | 35.6  Range 17-85 | 471mg | NR | 28.5% / 20 | No or poor therapeutic response 33.3% (5)  Patient refusal to take medication or have bloods 33.3% (5)  Excessive drowsiness 26.6% (4)  Haematological complications 20% (3)  Unknown 20% (3) | Poor | 1 |
| Ciapparelli A, 2003 | Prospective Naturalistic | 101 | Male 69%  Female 31% | NR | NR | NR | 54% / 54 | Inefficacy (15) 15%  Noncompliance (17) 17%  Side effects (5) 5%  Distance (13) 13%  Pregnancy (2) 2%  Improvement (2) 2% | Good | 3 |
| Davis MC, 2014 | Retrospective Naturalistic study | 320 | NR | 48 (0.6) | NR | Median 1.652 years    Range 0.405–5.57 years | 57% / 183 | Non Adherence (refusal or misuse of medication, elopement, failure to attend appointments, lack of cooperation with laboratory monitoring or refusal of treatment without specific reason) 35% (112)  Side effects 28% (90)  Administrative (discharge to non-DVAMC healthcare system or to a venue where clozapine treatment was unavailable or impractical) 19% (61)  Emergent Illness 20% (38)  Death 12% (24)  Treatment failure 4% (13)  Registered, never started 1% (3) | Good | 3 |
| Drew, 2002 | Retrospective cohort | 32 | NR | NR | NR | NR | 31% / 10 | Side effects (4) 40%  -cardiomyopathy 1  -cardiac arrythmia 1  -not recorded 2  Non compliance (6) 60% | Poor | 1 |
| Elachola M, 2025 | Retrospective | 30 | Male 67% Female 33% | 32.3 (9.78) | NR | NR | can only provide data for next column, ie % of reasons for dc, as we do not know the total treated population | Adverse effects 57% (17)  - Myoclonus/seizure n=4  - Neutropenia n=4  - Constipation n=1  - Sedation n=3  - Hyperglycaemia/metabolic syndrome n=3  - Myocarditis n=1  - Postural hypotension n=1  Inadequate therapeutic response 23% (7)  Non-Adherence 13% (4)  Patient/caregiver preferred to stop clozapine as blood monitoring and frequent visits to hospital was difficult 3% (1)  Expressed emotions 3% (1) | Fair | 2 |
| Ercis M, 2025 | Retrospective cohort | 46 | Male 71.7% Female 28.3% | NR | NR | NR | 48% / 22 | Adverse drug reaction 59% (13) - Myocarditis/pericarditis n=2 - Noncardiac inflammatory response n=1 - Pneumonia n=2 - Constipation n=3 - Orthostasis/dizziness/falls n=2 - Sedation n=3 - Sialorrhea n=3  Ineffective 9% (2) Patient choice / nonadherence 23%( 5) Other/Unknown 36% (8) | Good | 3 |
| Gee, 2018 | Retrospective cohort | 133 | Male 70%  Female 30% | Clozapine continuers 32 (12–70)  Clozapine discontinuers 30 (17–52) | NR | NR | 36% / 48 | Patient refusal (36) 75%  Blood dyscrasia (9)19% Medical reason (2) 4%  Change in diagnosis (1) 2% | Good | 3 |
| Groenewald Floor C. E, 2024 | Retrospective cohort | 284 | Male 69%  Female 31% | <55 group (n=183): 34.1 (26.6–44.7)  >55 group (n=101): 63 (58.0–70.7) | <55 age group:  300mg  >/= 55 age group:  200 | <55 group  Mean 3.975 years  Range 0.65–5.975 years  >/= 55 group  Mean1.492 years  Range 0.317–3.275  years  Mean age: 44.4 years (total population) | 2% / 65 | Sedation 48% (31)  Hypersalivation 28% (18)  Dizziness/orthostasis 14% (9)  Neutropenia 9% (6) | Good | 3 |
| Grover S, 2023 (a) | Retrospective cohort | 859 | NR | NR | NR | 1.68 (2.52) years | 5% / 44 (only including those who discontinued due to doctor led decision) | Blood dyscrasias 27.27% (12)  Poor adherence making the hematological monitoring difficult 20.45% (9) Intolerable sedation 15.9% (7) | Poor | 1 |
| Grover S, 2023 (b) | Cohort | 671 | Male 60.2%  Female 39.8% | 34.22 (11.65) | NR | Mean 1.88 (2.55) years    Median 1 year  Range 1–15.33 years | 26% / 176 | Long distance from the hospital 15.3% (27)  Long waiting time for consultation 4.5% (8)  No benefit with treatment 9.7% (17)  Side effects with medication 5.7% (10)  Moving away to another place 3.4% (6)  Refusal by the patient to follow-up 4% (7)  Patient improved and so did not feel the need to continue treatment 4% (7)  Other reasons 21% (37)  Unknown 52.3% (92) | Poor | 1 |
| Guo X, 2011 | Prospective observational cohort | 177 | Male 59.9%  Female 40.1% | 25.6 (7.2) | 266.5mg | Mean 0.767 (0.31) years | 36.7% / 65 | Relapse 27/65 41.5% (27)  Patient decision or lost to follow up 35.3% (23)  Intolerability 12.3% (8)  Other 10.8% (7) | Good | 3 |
| Haro J, 2007 | Prospective observational cohort study | 274 | Male 62.3%  Female 37.7% | 36.5 (10.2) | 239mg | Mean 0.875  (0.07) years | 55% / 151 | Lack of effectiveness 66.4% (100)  Intolerability 18.6% (28)  Lack of compliance 9.1% (14)  Patient request 16.4% (25) | Good | 3 |
| Hodgson R, 2005 | Retrospective cohort study | 44 | Male 81.8%  Female 18.2% | 37.3 (1.6) | 332.3mg | Median 6 years | 38.6% / 17 | Ineffective 6% (1)  Poor compliance 18% (3)  Side effects 12% (2)  Patient request 12% (2)  Serious adverse event 6% (1)  Other 24% (4)  Unknown 24% (4) | Fair | 2 |
| Imazu S, 2021 | Retrospective | 8263 | Male 54.1%  Female 45.9% | 40  Range 32-49 | 186.41mg | NR | 33% / 2716 | Efficacy 19% (508)  Safety 35% (954)  Other reasons 46% (1254) incl:  – cardiac disorder 1.5% (42)  – gastrointestinal disorder 3% (73)  – glucose intolerance 2% (59)  – NMS 0.6% (17)  – leukopenia 15% (411)  – pleurisy 0.2% (6)  – PE 0.1% (4)  – sedation 1% (29)  – seizure 1% (21) | Good | 3 |
| John AP, 2023 | Retrospective | 457 | Male 68.9%  Female 31.1% | 44.13 (13.58) | NR | Mean 4.541 (0.254) years | 70% / 318 | Nonadherence 48.9% (156)  Personal reasons 7.8% (25)  Family/carer objections 0.6% (2)  Cardiac complications (unspecified) 6.0% (19)  Myocarditis 5.0% (16)  Postural hypotension 0.9% (3)  Tachycardia 1.3% (4)  Sedation 1.9% (6)  Seizures 0.3% (1)  Weight gain 0.9% (3)  Neutropenia 4.4% (14)  Eosinophilia 0.3% (1)  Hypersalivation 0.3% (1)  Other adverse effects not listed 3.8% (12)  Other medical reasons not listed 12 3.8% (12)  Inadequate response, or no efficacy 3.4% (11)  Death 2.5% (8)  Switching clozapine brands 0.6% (2)  Interruption reason undetermined 6.9% (22) | Good | 3 |
| Kelly DL, 2024 | Prospective cohort | 274 | NR | 40.7 (11.5) | 271.9mg | NR | 17% / 47 | Prescriber choice 2% (1)  Participant choice 32% (15)  Lost to follow up 17% (8)  Nonadherence 13% (6)  Lack of therapeutic response 4% (2)  Serious adverse event 30% (14)  Administrative error 2% (1) | Good | 3 |
| Kelly DL, 2007 | Retrospective Cohort | 1875 | NR | NR | NR | NR | 46% / 864 | Lack of efficacy 23% (199)  Nonadherence 29% (247)  Agranulocytosis 1% (8)  Leucopenia 7% (62)  Other haematological adverse effects 0.7% (6) (thrombocytopenia, low complete blood count, eosinophilia)  Other nonhematologic adverse effects 13% (111)  Death 3% (29)  Other 7% (63)  Unknown 16% (139) | Fair | 2 |
| Kocyigit D, 2025 | Retrospective Cohort | 178 | NR | NR | 245mg | NR | 8% / 14 | Serious side effects 100% (14) | Good | 3 |
| Krivoy A, 2011 | Retrospective cohort | 100 | Male 61%  Female 39% | 35.3 (13.1) | 398mg | Mean 3.75 (2.83)  years | 42% / 42 | Nonadherence 47.6% (20)  Refusal to undergo repeated blood tests 21% (9)  Insufficient clinical response 19% (8)  Unacceptable side effects 26.2% (11) incl  - Electroencephalogram 12% (5)  - Severe constipation 9.5% (4)  - Somnolence 7% (3)  - Neutropenia 5% (2)  - Not specified 7% (3) | Good | 3 |
| Leclerc, 2021 | Retrospective | 29 | Male 89%  Female 11% | 21.1 (2.9) | NR | Mean 1.1887 (1.06) years Range 0.05–4.268 years | 7% / 2 | Neutropenia (2) 100% | Poor | 1 |
| Legge SE, 2016 | Retrospective cohort | 316 | Male 65%  Female 35% | 36.23 (10.9) | NR | Mean 0.492 years  Median 0.33 years | 45% / 142 | ADR - 56% (80)  Non adherence NOS 18% (25)  Blood monitoring 8% (11)  Inadequate response 6% (8)  Belief medication not required 3% (4)  Delusional belief 3% (4)  Anticipated non adherence 1.4% (2)  Other 3.5% (5)  Death 2% (3) | Good | 3 |
| Leppig M, 1989 | Retrospective cohort | 121 | Male 37%  Female 63% | 42 (16) | 131mg | Mean 2.667 (3.583 )  years | 27% / 33 | Refusal of treatment 36.4% (12)  Insufficient therapeutic effect 33.3% (11)  Psychological dependency of clozapine, non-compliance, pregnancy 3% (1)  Side effects 21% (7) - Hypotension and fatigue 12% (4) - Weight gain 6% (2)  - Delirious state (patient also received amitriptyline) 3% (1) | Poor | 1 |
| Lindstrom, 1988 | Retrospective cohort | 96 | Male 67%  Female 33% | 36.1 | NR | NR | 36% / 35 | Inadequate therapeutic effect 31% (11) Lack of compliance 20% (7)  Withdrawn from the market in 1975 20% (7)  Sedation 6% (2)  Hyperthemia 3% (1)  Grand mal seizures 3% (1)  Dizziness 3% (1)  Problems with blood controls 3% (1)  Underwent psychotherapy 3% (1)  Improvement, free from symptoms 3% (1)  Leukopenia 3% (1)  Agranulocytosis 3% (1) | Fair | 2 |
| MacGillivary S, 2003 | Retrospective cohort | 201 | Male 63%  Female 37% | 40.5  Range 17-75 | NR | NR | 26% / 52 | No improvement 33% (17)  Non-compliance (to either blood monitoring or taking tablets) 13% (7)  Side effects 17% (9)  Clozapine monitoring service alert 17% (9)  Other (e.g death) 10% (5)  Unknown 10% (5) | Good | 3 |
| MacPherson R, 1998 | Retrospective Cohort | 19 | NR | NR | NR | NR | 21% / 4 | No response 25% (1)  Non-fatal neuroleptic malignant syndrome 25% (1)  Recurrent chest infection plus non response 25% (1)  Fainting, hypotension, episodes of unconsciousness, seizures and failing to improve 25% (1) | Poor | 1 |
| Martin A, 2008 | Retrospective | 47 | NR | NR | 514mg | NR | 32% / 15 | Not documented 13% (2)  Sedation 13% (2)  Weight gain 7% (1)  Patient refusal (poor insight) 13% (2)  Seizure 7% (1)  Hypersalivation 7% (1)  Hyperglycaemia 7% (1)  Neutropenia 13% (2)  Ineffective 7% (1)  Not documented in the paper 13% (2) | Fair | 2 |
| Moeller FG, 1995, | Retrospective Cohort | 805 | Male 95%  Female 5% | 42.9 (8.6) | NR | NR | 21% / 167 | Administrative issues 44% (73)  Side effects 28% (46)  Lack of Response 21% (36)  Patient Death 5% (8)  Concomitant medical illness 2% (3) | Fair | 2 |
| Mustafa FA, 2014 | Retrospective | 190^a^ | Male 64.2%  Female 35.8% | 46  Range 22-77 | NR | NR | can only provide data for next column, ie % of reasons for dc, as we do not know the total treated population | Non Compliance (with treatment or mandatory blood monitoring) 55.3% (105)  Neutropenia 14.2% (27)  Other adverse effects 11% (21)  Death 10% (19)  Physician’s decision 5.3% (10)  Inadequate therapeutic response n = 3.2% (6)  Not recorded 1% (2) | Good | 3 |
| O’Connor D, 2010 | Retrospective | 75 | Male 37.3% Female 62.7% | 74.2 (Range 65-89) | 296mg | Mean 4.45 years Range 1-14 years | 49% / 37 | Death 38% (14)  - none could be attributed unequivocally to clozapine, though orthostatic hypotension might possibly have contributed to a death due to stroke  Adverse events 32% (12)  - Orthostatic Hypotension n=2  - Fever, tachycardia n=2  - “Red” WBC n=2  - Myocarditis, myocardial infarction, “red” WBC n=1  - Myocardial infarction n=1  - Cardiomyopathy n=1  - Sedation, dysphagia n=1  - Sedation, ataxia n=1  - Parkinsonism n=1  Patient choice 22% (8) [refused treatment or failed to adhere to monitoring protocol]  Physical frailty 5% (2)  Unknown 3% (1) | Fair | 2 |
| Ord KL, 2023 | Retrospective | 102 | Male 67.6%  Female 32.4% | Range 18-62 | 350mg | Mean 0.229 (0.106) years  Range 0.057-0.384 years | 14% / 14 | Tachycardia 28.6% (4)  Neutropenia 21.4% (3)  Constipation 7.1% (1)  Nocturnal enuresis and hypersalivation 7.1% (1)  Worsening OCD symptoms 7.1% (1)  Ileus 7.1% (1)  No longer clinically indicated 7.1% (1)  No clinical response in the treatment of tardive dyskinesia 7.1% (1)  No clinical response in the treatment of psychosis with a combination of intolerable side effects: Constipation, hypersalivation, sedation and tachycardia 7.1% (1) | Fair | 2 |
| Pai NB, 2012 | Retrospective | 151^b^ | Male 68%  Female 32% | 37.12 (12.56) | NR | NR | can only provide data for next column, ie % of reasons for dc, as we do not know the total treated population | Non-compliance 36% (54)  Own decision 40% (60)  Medical 17% (26)  Poor response 3% (4)  Other (e.g patient missing during treatment, amotivation, disliking blood tests) 4% (6) | Poor | 1 |
| Rascati KL, 1993 | Retrospective | 852 | Male 65.1%  Female 34.9% | 38 (9)  Range 18-70 | NR | Mean 0.392 (0.417) years  Range 0-2.417 years  Median 0.25  years | 16% / 134 | No clinical response 22.4% (30)  Patient refusal or request 20.1% (27)  Adverse effects other than low leukocyte count 16.4% (22)  Low leukocyte count 11.9% (16)  Patient no-show 7.5% (10)  Medication noncompliance 5.2% (7)  Other 7. 5% (10)  No reason reported 9%(12) | Poor | 1 |
| Rowntree R, 2020 | Retrospective cohort study | 31 | Male 75%  Female 25% | 28.44 | 380mg | Mean 1.6 (1.99) years  Range 0.17–7.5  years | 58% / 18 | Self discontinuation 55% (10)  Severe side effects 27.8% (5)  Severe sepsis 5.5% (1)  Agranulocytosis 5.5% (1)  Diagnostic change 5.5% (1) | Good | 3 |
| Sajatovic M, 1997 | Retrospective | 10 | NR | 70.6 (3.3) Range 65-76 | 204mg | Mean 1.178 (2.036)  Range 0.0356±66.23 years | 70% / 7 | Inability to comply with blood work 28.6% (2)  Lack of clinical improvement 42.9% (3)  Developed atrial fibrillation 14.3% (1)  Developed SLE 14.3% (1) | Poor | 1 |
| Sajatovic, 2000 | Retrospective cohort | 2996 | Male 95%  Female 5% | 44.8 (10.2) Range 21-95 | 503mg   (SD = 204, Range 25–900) | Mean 0.5±0.6 years   Range 0.153-4.7 years | 50% / 1486 | Noncompliance 12% (359)  Poor response 8% (235)  Administrative 8% (233)  Cardiovascular 1% (42)  Dizziness/sedation 1% (43)  Seizures 0.5% (14)  Neutropenia 2% (61)  Agranulocytosis 0.5% (14)  Eosinophilia 0.2% (7)  Death 1% (38)  Not recorded (440) | Poor | 1 |
| Shaker, 2018 | Retrospective | 25 -  only reported dis- continuers | Male 72%  Female 28% | 25.8 (4.6)  Range 19–35 | 404.5 mg   (SD = 169.9, Range 150–950) | 1.456 (1.246) years  Range 0.258–4.323 years | 25 | Non-compliance 44% (11)  Agranulocytosis/neutropenia 4% (1)  Other adverse effects 36% (9)  Patient decision 16% (4) | Fair | 2 |
| Taylor DM, 2009 | Case-control | 368* | NR | 40.0 (12.6)  Range 18–83 | 360mg | Mean 1.025 (1.55) years  Range 0.02–8.33 years  Median 0.25  years | 44% / 161 | Patient’s decision (partial or non adherence, or patient request or refusal) 47.8% (77)  Adverse effects (clinician decision to withdraw because of unacceptable side effects) 35.4% (57)  Ineffective (clinician assessment of inadequate effect) 1.9% (3)  Death 13% (21)  Other 1.9% (3) | Good | 3 |
| Thien K, 2018 | Retrospective cohort | 41 | NR | 19.5 (2.9)  Range 15-24 | NR | NR | 24% / 10 | Cardiac complications 60% (6)  Non Compliance 40% (4) | Fair | 2 |
| Ucok, 2019 | Retrospective | 396 | Male 65%  Female 35% | 38.2 (11.5) | 423.5mg ± 189.8mg | 4.5 ± 4.7 years | 156 | Side effects 49% (77)  -Leukopenia 15  -Sedation 12  -Seizure/myoclonic jerks 10  -Hypersalivation 7  -Incontinence 6  -Tachycardia 6  -Myocarditis 4  -Obsessive-compulsive symptoms 4  -Weight gain 3  -Diabetes mellitus and other metabolic disorders 2  -Orthostatic hypotension 2  -Other reasons 5  Non compliance (46) 30%  Inefficacy (33) 21% | Fair | 2 |
| Woerner MG, 2003 | Prospective cohort | 34 | Male 62%  Female 38% | 23 (5.2)  Range16-45 | 206mg | NR | 76% / 26 | Low WBC 23% (6)  Adverse effects (rash, urinary retention, weight gain) 11.5% (3)  Refused blood tests 19.2% (5)  Refusal of medication 34.6% (9)  Lack of improvement 11.5% (3) | Fair | 2 |
| Zito J, 1993 | Prospective cohort | 202 | Male 73% Female 27% | NR | NR | NR | 29% / 59 | Adverse effects 29% (17)  Poor response 48% (28)  Patient refusal 14% (8)  Diagnosis of cancer 2% (1)  Unknown 9% (5) | Fair | 2 |

Supplementary Table 4 - The Study Quality Assessment Tool developed by the National Heart, Lung and Blood Institute (NHLBI)

Quality of Assessment Tool for Cohort and Cross Sectional Studies

1. Was the research question or objective in this paper clearly stated?

2. Was the study population clearly specified and defined?

3. Was the participation rate of eligible persons at least 50%?

4. Were all the subjects selected or recruited from the same or similar populations (including the same time period)? Were inclusion and exclusion criteria for being in the study prespecified and applied uniformly to all participants?

5. Was a sample size justification, power description, or variance and effect estimates provided?

6. For the analyses in this paper, were the exposure(s) of interest measured prior to the outcome(s) being measured?

7. Was the timeframe sufficient so that one could reasonably expect to see an association between exposure and outcome if it existed?

8. For exposures that can vary in amount or level, did the study examine different levels of the exposure as related to the outcome (e.g., categories of exposure, or exposure measured as continuous variable)?

9. Were the exposure measures (independent variables) clearly defined, valid, reliable, and implemented consistently across all study participants?

10. Was the exposure(s) assessed more than once over time?

11. Were the outcome measures (dependent variables) clearly defined, valid, reliable, and implemented consistently across all study participants?

12. Were the outcome assessors blinded to the exposure status of participants?

13. Was loss to follow-up after baseline 20% or less?

14. Were key potential confounding variables measured and adjusted statistically for their impact on the relationship between exposure(s) and outcome(s)?

|  | **Q1** | **Q2** | **Q3** | **Q4** | **Q5** | **Q6** | **Q7** | **Q8** | **Q9** | **Q10** | **Q11** | **Q12** | **Q13** | **Q14** | **Overall Rating** | **Score** |
| --- | --- | --- | --- | --- | --- | --- | --- | --- | --- | --- | --- | --- | --- | --- | --- | --- |
| **Atkinson JM, 2007** | + | + | + | + | - | + | + | NA | + | - | + | - | + | NR | Good | 3 |
| **Baker White, 2004** | + | + | + | + | - | + | + | - | + | - | + | - | + | - | Fair | 2 |
| **Buoli M, 2016** | + | + | + | + | - | + | + | + | + | - | + | NA | - | + | Good | 3 |
| **Chen EYH, 1996** | + | + | + | + | - | + | + | - | - | - | + | - | + | + | Fair | 2 |
| **Chow EW, 1995** | - | - | CD | - | - | + | + | + | + | + | - | - | + | - | Poor | 1 |
| **Ciapparelli A, 2003** | + | + | + | + | - | + | + | + | + | + | + | - | + | + | Good | 3 |
| **Davis MC, 2014** | + | + | + | + | - | + | + | + | + | + | + | - | + | + | Good | 3 |
| **Drew LRH, 2002** | - | + | + | - | - | + | + | + | + | + | + | - | - | - | Poor | 1 |
| **Elachola M, 2025** | + | + | + | - | - | + | + | - | + | - | + | - | + | - | Fair | 2 |
| **Ercis M, 2025** | + | + | + | + | - | + | + | + | + | - | + | - | + | + | Good | 3 |
| **Gee SH, 2018** | + | + | + | + | - | + | + | - | + | + | + | - | + | + | Good | 3 |
| **Groenewald FCE, 2024** | + | + | + | + | - | + | + | + | + | + | + | - | + | + | Good | 3 |
| **Grover S, 2023 (a)** | + | - | CD | CD | - | + | NR | - | + | NR | + | - | + | + | Poor | 1 |
| **Grover S, 2023 (b)** | + | + | - | + | - | + | + | - | + | - | + | - | - | - | Poor | 1 |
| **Guo X, 2011** | + | + | + | + | + | + | + | + | + | + | + | - | - | + | Good | 3 |
| **Haro JM, 2007** | + | - | + | + | - | + | + | + | + | + | + | - | - | + | Good | 3 |
| **Hodgson R, 2005** | - | + | + | + | - | + | + | - | + | - | + | - | + | + | Fair | 2 |
| **Imazu S, 2021** | + | + | + | + | - | + | + | + | + | - | + | - | + | - | Good | 3 |
| **John AP, 2023** | + | + | + | + | - | + | + | + | + | + | + | - | + | + | Good | 3 |
| **Kelly DL, 2024** | + | + | + | + | - | + | + | + | + | + | + | - | + | NR | Good | 3 |
| **Kelly DL, 2007** | + | + | + | + | - | + | + | - | + | - | + | - | - | + | Fair | 2 |
| **Kocyigit D, 2025** | + | + | + | + | - | + | + | - | + | + | + | - | + | - | Good | 3 |
| **Krivoy A, 2011** | + | + | + | + | - | + | + | + | + | + | + | - | + | + | Good | 3 |
| **Leclerc LD, 2021** | + | + | CD | + | - | + | + | - | + | - | + | - | NR | - | Poor | 1 |
| **Legge SE, 2016** | + | + | - | + | - | + | + | + | + | NA | + | NA | + | + | Good | 3 |
| **Leppig M, 1989** | - | - | CD | NR | - | + | CD | + | + | CD | - | - | NA | - | Poor | 1 |
| **Lindström LH, 1988** | + | + | + | + | - | + | + | - | + | - | - | - | + | - | Fair | 2 |
| **MacGillivray S, 2003** | + | + | + | + | - | + | + | NA | + | - | + | NA | - | + | Good | 3 |
| **MacPherson R, 1998** | - | - | + | - | - | + | + | - | + | - | - | - | + | NA | Poor | 1 |
| **Martin A, 2008** | + | + | + | + | - | + | + | - | + | - | + | - | + | - | Fair | 2 |
| **Moeller FG, 1995** | + | + | NR | + | NR | + | + | + | + | - | - | - | + | + | Fair | 2 |
| **Mustafa FA, 2014** | + | + | + | + | - | + | + | - | + | - | + | - | + | + | Good | 3 |
| **O’Connor D, 2010** | + | + | + | + | - | + | + | - | + | - | + | - | + | - | Fair | 2 |
| **Ord KL, 2023** | + | + | + | + | - | + | + | + | + | - | + | - | NA | - | Fair | 2 |
| **Pai NB, 2012** | + | - | CD | - | - | + | + | + | + | - | + | - | NA | - | Poor | 1 |
| **Rascati KL, 1993** | + | + | + | + | - | + | + | - | + | - | - | - | + | - | Poor | 1 |
| **Rowntree R, 2020** | + | + | + | + | - | + | + | + | + | - | + | - | + | - | Good | 3 |
| **Sajatovic M, 2000** | + | - | CD | - | - | + | - | + | - | + | + | - | - | + | Poor | 1 |
| **Shaker, 2018** | + | + | + | + | - | + | + | - | + | - | + | - | + | + | Fair | 2 |
| **Thien K, 2018** | + | + | + | + | - | + | + | - | + | - | + | - | + | - | Fair | 2 |
| **Ucok A, 2019** | + | - | CD | - | - | + | + | + | + | + | + | - | + | + | Fair | 2 |
| **Woerner MG, 2003** | + | + | CD | + | - | + | + | + | + | + | + | NR | + | - | Fair | 2 |
| **Zito J, 1993** | + | + | + | NR | - | + | + | + | - | + | + | - | - | + | Fair | 2 |

CD = Cannot determine

NA = Not applicable

NR = Not reported

Quality of Assessment Tool for Before-After Studies with No Control Group

1. Was the study question or objective clearly stated?

2. Were eligibility/selection criteria for the study population prespecified and clearly described?

3. Were the participants in the study representative of those who would be eligible for the test/service/intervention in the general or clinical population of interest?

4. Were all eligible participants that met the prespecified entry criteria enrolled?

5. Was the sample size sufficiently large to provide confidence in the findings?

6. Was the test/service/intervention clearly described and delivered consistently across the study population?

7. Were the outcome measures prespecified, clearly defined, valid, reliable, and assessed consistently across all study participants?

8. Were the people assessing the outcomes blinded to the participants' exposures/interventions?

9. Was the loss to follow-up after baseline 20% or less? Were those lost to follow-up accounted for in the analysis?

10. Did the statistical methods examine changes in outcome measures from before to after the intervention? Were statistical tests done that provided p values for the pre-to-post changes?

11. Were outcome measures of interest taken multiple times before the intervention and multiple times after the intervention (i.e., did they use an interrupted time-series design)?

12. If the intervention was conducted at a group level (e.g., a whole hospital, a community, etc.) did the statistical analysis take into account the use of individual-level data to determine effects at the group level?

|  | **Q1** | **Q2** | **Q3** | **Q4** | **Q5** | **Q6** | **Q7** | **Q8** | **Q9** | **Q10** | **Q11** | **Q12** | **Overall Rating** | **Score** |
| --- | --- | --- | --- | --- | --- | --- | --- | --- | --- | --- | --- | --- | --- | --- |
| **Sajatovic M, 1997** | + | + | + | + | - | + | - | - | + | - | + | CD | Poor | 1 |

CD = Cannot determine

Quality of Assessment Tool for Case Control Studies

1. Was the research question or objective in this paper clearly stated and appropriate?

2. Was the study population clearly specified and defined?

3. Did the authors include a sample size justification?

4. Were controls selected or recruited from the same or similar population that gave rise to the cases (including the same timeframe)?

5. Were the definitions, inclusion and exclusion criteria, algorithms or processes used to identify or select cases and controls valid, reliable, and implemented consistently across all study participants?

6. Were the cases clearly defined and differentiated from controls?

7. If less than 100 percent of eligible cases and/or controls were selected for the study, were the cases and/or controls randomly selected from those eligible?

8. Was there use of concurrent controls?

9. Were the investigators able to confirm that the exposure/risk occurred prior to the development of the condition or event that defined a participant as a case?

10. Were the measures of exposure/risk clearly defined, valid, reliable, and implemented consistently (including the same time period) across all study participants?

11. Were the assessors of exposure/risk blinded to the case or control status of participants?

12. Were key potential confounding variables measured and adjusted statistically in the analyses? If matching was used, did the investigators account for matching during study analysis?

|  | **Q1** | **Q2** | **Q3** | **Q4** | **Q5** | **Q6** | **Q7** | **Q8** | **Q9** | **Q10** | **Q11** | **Q12** | **Overall Rating** | **Score** |
| --- | --- | --- | --- | --- | --- | --- | --- | --- | --- | --- | --- | --- | --- | --- |
| **Taylor DM, 2009** | + | + | - | + | + | + | - | + | + | + | - | - | Good | 3 |

NR = Not reported

| **Supplementary Table 5** Subgroup Analysis of Clozapine Discontinuation | | | | | | | |
| --- | --- | --- | --- | --- | --- | --- | --- |
| Analysis | Meta-Analysis | | | | Heterogeneity | | |
|  | Pooled Prevalence | Mean (%) | 95% CI | between group differences p-value | I² | Q value | p value |
| Sample Size | 36.5 (31.2-41.9) | <131: 36.4 | 29.1-44.1 | 0.974 | 98.03 | 1415.33 | <0.001 |
|  |  | ≥131: 36.5 | 31.2-44.4 |  |  |  |  |
| Age | 38.3 (32.2-44.6) | <38.4: 34.5 | 27.0-42.4 | 0.373 | 98.15 | 1158.05 | <0.001 |
|  |  | ≥38.4: 41.6 | 31.4-52.1 |  |  |  |  |
| Study Year | 36.5 (31.2-41.9) | <2011: 38.2 | 31.8-44.8 | 0.474 | 98.03 | 1415.33 | <0.001 |
|  |  | ≥2011: 34.2 | 31.2-41.9 |  |  |  |  |
| Study Design | 36.5 (31.2-41.9) | Retro: 35.1 | 29.2-41.3 | 0.109 | 98.03 | 1415.33 | <0.001 |
|  |  | Pros: 40.9 | 28.2-54.3 |  |  |  |  |
|  |  | CC: 43.7 | 38.7-48.9 |  |  |  |  |
| Region | 36.5 (31.2-41.9) | EU: 39.7 | 27.6-52.5 | 0.506 | 98.03 | 1415.33 | <0.001 |
|  |  | NA: 36.5 | 24.4-49.4 |  |  |  |  |
|  |  | ROW: 33.0 | 31.2-41.9 |  |  |  |  |
| Clozapine Duration | 38.3 (29.5-47.5) | <6 mos: 25.2 | 7.1-49.5 | 0.207 | 98.31 | 788.15 | <0.001 |
|  |  | ≥6 mos: 41.5 | 32.4-50.9 |  |  |  |  |
| Clozapine Dose | 37.3 (29.7-45.2) | <350mg: 36.8 | 25.5-48.9 | 0.838 | 98.04 | 539.47 | <0.001 |
|  |  | ≥350mg: 38.5 | 29.7-45.2 |  |  |  |  |
| Quality Assessment | 34.3 (28.7-40.3) | Good: 39.9 | 31.7-48.7 | 0.136 | 98.43 | 1489.04 | <0.001 |
|  |  | Fair: 34.5 | 26.7-43.3 |  |  |  |  |
|  |  | Poor: 24 | 14.3-37.4 |  |  |  |  |

Retro = Retrospective, Pros = Prospective, CC = Case-control, EU = Europe, NA = North America, ROW = Rest of World, mos = months, mg = milligram

| **Supplementary Table 6** Meta Regression of Clozapine Discontinuation | | | | | | |
| --- | --- | --- | --- | --- | --- | --- |
| Main analysis | | Number of  comparison | β | 95% CI | | p-value |
| Moderator | |  |  |  |  |  |
|  | Study Year | 39 | -0.0011 | -0.0113 | 0.0092 | 0.835 |
|  | Sample Size | 39 | 6.96x10^-6^ | -0.0007 | 0.00008 | 0.900 |
|  | Mean Age | 28 | 0.0089 | -0.0021 | 0.0198 | 0.114 |
|  | Clozapine Dose | 20 | 0.0004 | -0.0010 | 0.0019 | 0.562 |
|  | Clozapine Duration | 17 | 0.0792 | -0.0243 | 0.1826 | 0.134 |

Supplementary Table 7 – Deaths

| Study | Number of Deaths | Reasons for Death |
| --- | --- | --- |
| Atkinson 2007 | 12 | Seizure n=2  Unknown n=2  Overdose of Clozapine n=1  Perforated gastric ulcer n=1  Pulmonary embolism n=1  Chronic bowel obstruction n=1  Lung carcinoma n=1  Stroke n=1  Left Ventricular failure due to hypertrophy n=1  Bronchial pneumonia n=1 |
| Davis 2014 | 24 (two attributed to clozapine) | Agranulocytosis n=1  Obstipation n=1  Cardiovascular n=14  Pulmonary n=3  Not documented n=5 |
| John 2023 | 8 | The study reports they didn’t have access to cause of death but all noted to have occurred after the first 18 weeks of treatment |
| Kelly 2007 | 29 | The study didn’t report on cause of death and didn’t comment if any deaths were attributed to clozapine. |
| Legge 2016 | 3 | The study didn’t report on cause of death but noted to include death as a reason for discontinuation regardless of whether the cause was attributed to clozapine. |
| MacGillivary 2003 | 5 | The study didn’t report on cause of death. They categorised reasons for discontinuation with one category as ‘Other (eg death)’ so therefore all five cases in this category may not be related to death. |
| Moeller 1995 | 8 | Bowel infarction n=1  Renal failure n=1  Pneumonia n=3  Suicide n=2  Sudden death of unknown aetiology n=1 |
| Mustafa 2014 | 19 | Respiratory infection n=5  Not documented n=5  Faecal impaction n=2  Suicide n=1  Alcohol intoxication n=1  Brain abscess n=1  Cancer n=1  Acute pancreatitis n=1  Pulmonary embolism n=1  Suspected clozapine Intoxication n=1 |
| O’Connor 2010 | 14 | None could be attributed unequivocally to clozapine, though orthostatic hypotension might possibly have contributed to a death due to stroke. |
| Taylor 2009 | 21 | Pneumonia n=5  Lung carcinoma n=3  Other carcinoma n=2  Myocardial infarction n=2  Cerebrovascular accident n=2  Clozapine overdose n=2  Gastrointestinal haemorrhage n=1  Cardiac arrest n=1  Left ventricular failure n=1  Asphyxia during restraint n=1  Sepsis n = 1 |
| Sajatovic 2000 | 38 | Most deaths were due to underlying medical illness n=29 - Accidental injury n=4 - Unclear etiology n=11 - Agranulocytosis n=2 - Suicide n=2 The study reports that agranulocytosis deaths were not causally related to any deviations in clozapine treatment or management protocols. |

Supplementary Figure 1 - Pooled Prevalence of Discontinuation Due to Adverse Effects Among Clozapine Discontinuers

Supplementary Figure 2 - Pooled Prevalence of Discontinuation Due to Non-Adherence Among Clozapine Discontinuers

Supplementary Figure 3 - Pooled Prevalence of Discontinuation Due to Ineffectiveness Among Clozapine Discontinuers

Supplementary Figure 4 - Clozapine discontinuation: funnel plot of standard error by proportion in quantitative synthesis of discontinuation
